# Attention Bias Modification (ABM): Review of Effects of Multisession ABM Training on Anxiety and Threat-Related Attention in High-Anxious Individuals

**DOI:** 10.1177/2167702617696359

**Published:** 2017-04-26

**Authors:** Karin Mogg, Allison M. Waters, Brendan P. Bradley

**Affiliations:** 1 Psychology, University of Southampton; 2 School of Applied Psychology, Grifﬁth University

**Keywords:** anxiety, attention bias modification, threat, cognitive control

## Abstract

Attention bias modification (ABM) aims to reduce anxiety by reducing attention bias (AB) to threat; however, effects on anxiety and AB are variable. This review examines 34 studies assessing effects of multisession-ABM on both anxiety and AB in high-anxious individuals. Methods include ABM-threat-avoidance (promoting attention-orienting away from threat), ABM-positive-search (promoting explicit, goal-directed attention-search for positive/nonthreat targets among negative/threat distractors), and comparison conditions (e.g., control-attention training combining threat-cue exposure and attention-task practice without AB-modification). Findings indicate anxiety reduction often occurs during both ABM-threat-avoidance and control-attention training; anxiety reduction is not consistently accompanied by AB reduction; anxious individuals often show no pretraining AB in orienting toward threat; and ABM-positive-search training appears promising in reducing anxiety. Methodological and theoretical issues are discussed concerning ABM paradigms, comparison conditions, and AB assessment. ABM methods combining explicit goal-directed attention-search for nonthreat/positive information and effortful threat-distractor inhibition (promoting top-down cognitive control during threat-cue exposure) warrant further evaluation.

Attention bias modification (ABM) training offers computer-delivered treatment for anxiety. Its development was based on the view that anxious individuals are characterized by a bias to selectively attend to threat cues in their environment and this attention bias (AB) toward threat plays a causal role in anxiety; hence, ABM training methods that reduce AB to threat should reduce anxiety (MacLeod & Clarke, 2015; MacLeod & Mathews, 2012; MacLeod, Rutherford, Campbell, Ebsworthy, & Holker, 2002). The most widely used ABM method is *ABM-threat-avoidance training*. This typically employs the modified visual-probe task (described later), which is designed to reduce AB in orienting toward threat cues (e.g., Amir, Beard, Burns, & Bomyea, 2009; Amir, Beard, Taylor et al., 2009; Heeren, Mogoase, McNally, Schmitz, & Philippot, 2015; MacLeod et al., 2002; McNally, Enock, Tsai, & Tousian, 2013). It requires participants to repeatedly respond to a probe (e.g., small dot, or letter) that appears in a different location to that just occupied by a threat cue, using implicit training to modify the direction of attention-orienting responses away from threat (Bar-Haim, 2010). Another less frequently used method is *ABM-positive-search training*, which explicitly encourages participants to search for positive/nonthreat target cues, which are embedded in picture-arrays of task-irrelevant negative/threat cues (e.g., Dandeneau, Baldwin, Baccus, Sakellaropoulo, & Pruessner, 2007; de Voogd, Wiers, Prins, & Salemink, 2014; Waters, Pittaway, Mogg, Bradley, & Pine, 2013; Waters et al., 2015, 2016).

Although early studies using ABM-threat-avoidance training with clinically anxious individuals were promising (e.g., Amir, Beard, Burns et al., 2009; Amir, Beard, Taylor et al., 2009), replication failures and meta-analyses indicate inconsistent effects on anxiety and question its clinical utility (e.g., see reviews and meta-analyses by Beard, Sawyer, & Hofmann, 2012; Cristea, Kok, & Cuijpers, 2015; Emmelkamp, 2012; Heeren, Mogoase, Philippot et al., 2015; MacLeod & Clarke, 2015; Mogoase, David, & Koster, 2014). Moreover, the efficacy of ABM-threat-avoidance training depends on its mode of delivery; that is, it tends to be more effective in reducing anxiety relative to control conditions when delivered in laboratory settings but not when delivered at home, which constrains its therapeutic usefulness and prevents widespread dissemination (Cristea et al., 2015; MacLeod & Clarke, 2015). Recommendations from these reviews differ. Cristea et al. (2015) discouraged further fine-grained research to analyze permutations of ABM methods that may influence its efficacy. Instead, they advocated large-scale randomized controlled trials (RCTs) using other cognitive bias modification methods that show promise in treating emotional disorders.

MacLeod and Clarke (2015) instead identified two questions for ABM research: (1) Is the attention-training method capable of reducing selective attention to threat? (2) If so, does it reduce anxiety? They tabulated findings from single-session and multisession ABM studies assessing effects of ABM on both AB and anxiety; these included 22 multisession ABM studies, 18 of which were RCTs of high-anxious individuals using ABM with threat cues (17 ABM-threat-avoidance studies and one ABM-positive-search training).^1^ The summary table indicated whether for each study (a) *ABM was achieved* and (b) *anxiety symptom change was observed*. This suggested a strong correlation between these outcomes. Of 22 multisession studies, 45% indicated change in both AB and anxiety during ABM training relative to control conditions, whereas the others showed no difference in change in either measure. One interpretation of these results is that, for ABM interventions to be effective in reducing anxiety, they have to be effective in reducing AB to threat.

However, modification of preexisting AB to threat may not fully explain anxiety reduction during ABM training for several reasons. First, AB to threat (typically assessed on probe-based attention tasks) has not been consistently observed in anxious individuals before training (Heeren, Mogoase, Philippot et al., 2015). Second, anxiety reduction may occur during ABM training without AB reduction. Third, other processes may contribute to the anxiolytic effect of ABM training, such as improvement in attention control (e.g., Heeren, Mogoase, McNally et al., 2015; McNally et al., 2013; see also Bar-Haim, 2010; Chen, Clarke, Watson, MacLeod, & Guastella, 2015). Top-down attention control processes may also be modified by control training conditions, thereby contributing to evidence of anxiety reductions in both active ABM as well as control conditions (discussed later).

The major aim of this review is to examine the methodology and outcomes of RCTs that assessed effects of multisession ABM training on both anxiety and AB in high-anxious individuals and to consider their theoretical and research implications. In common with MacLeod and Clarke (2015), we conducted a qualitative review to allow detailed examination of variations in methodology and findings across studies that may be obscured in a quantitative review (Cristea et al., 2015). For example, meta-analyses commonly treat AB as a unitary construct and do not discriminate between differing ABM training methods and AB indices. However, this conceptual view of AB is challenged by recent cognitive models of anxiety (see Mogg & Bradley, 2016, for a review), and ABM studies use a variety of AB measures (e.g., differing indices of ABs in attention orienting and threat-distractor interference, AB variability), which have implications for the interpretation of results, replication efforts, and the development of more effective ABM methods (discussed later).

Moreover, the present review complements and extends prior reviews in several ways: (1) It focuses only on *multisession RCTs* that randomly allocated *high-anxious* individuals (i.e., those with high subclinical or diagnosed anxiety) to threat-related ABM training or control conditions and assessed changes in *both* anxiety symptoms and AB. These studies have greater translational relevance to therapeutic application than studies evaluating single-session ABM and unselected participant samples. (2) It examines findings from a *larger number of RCTs* of multisession-ABM training of high-anxious individuals than prior reviews (*N* = 34 studies, only 18 of which were included in MacLeod and Clarke’s, 2015, review). (3) It *distinguishes* between ABM-threat-avoidance and ABM-positive-search training because these interventions differ in several ways, discussed later. (4) It considers effects of *control training* on anxiety and AB and implications for evaluating ABM efficacy. (5) It examines *methodological issues* in AB assessment and modification, which influence the interpretation of findings of AB change. (6) It considers the *implications* of differing theoretical views of anxiety for ABM research. Together, these issues are important in guiding decision-making about experimental designs and methodology in future ABM research, such as the choice of ABM intervention, comparison conditions, and AB assessment methods.

To identify studies for inclusion in this review, we searched PsycInfo and Medline databases of peer-reviewed English-language research publications (search terms: anx* and “attention* bias modification” or “attention* bias training”) and checked reference lists of recent empirical and review papers for additional articles. Studies that met each of the following inclusion criteria were selected for this review: (1) the study used multisession-ABM training with the aim of modifying AB to threat and anxiety; (2) ABM training used threat and nonthreat stimuli; (3) participants were high-anxious individuals with subclinical or clinical anxiety; (4) the study employed an RCT design, which randomly allocated high-anxious participants to ABM or control conditions (e.g., waitlist, control training); (5) there was assessment of change in anxiety symptoms from pre- to posttraining; and (6) there was assessment of change in AB from pre- to posttraining. Review articles were excluded as well as empirical studies that used a combination of ABM and interpretive bias modification training (where outcomes could not be attributed specifically to ABM). Consequently, we identified 34 studies published between 2008 and the end of 2016 that met the specifications for this review (33 publications, with one describing two studies; indicated in “References”).^2^ Of these, 32 studies used ABM-threat-avoidance training and two used ABM-positive-search training. We evaluate their findings against the main predictions on which the studies were based: (1) There will be greater reduction in anxiety symptoms between pre- and posttraining for ABM training than for comparison conditions, and (2) there will be a corresponding pattern of reduction in AB to threat in these conditions. In addition, we consider evidence for the assumption of ABM-threat-avoidance training that anxious individuals show increased AB towards threat relative to nonthreat stimuli before training (Prediction 3), as this is a precondition of Prediction 2.

## Methods Used in ABM Training

### ABM-threat-avoidance training

Most ABM-threat-avoidance studies used the visual-probe task to reduce AB to threat (e.g., Amir, Beard, Burns et al., 2009). On each trial, two cues appear simultaneously in different regions of a computer screen, typically for 500 ms. On critical trials, one cue is threat-related (e.g., angry face) and the other nonthreat (e.g., neutral face). A target-probe (e.g., dot or letter) then replaces one of the cues, either in the location just occupied by the threat cue (threat-congruent trial) or opposite location (threat-incongruent trial). Participants respond as quickly as possible to the probe (e.g., indicate its location or identity). In ABM-threat-avoidance training, *probes are more likely to appear in the opposite location to threat cues* (i.e., threat-incongruent trials are more frequent than threat-congruent trials) to encourage attention orienting away from the spatial location in which a threat cue has just appeared. It aims to change AB by habit change from repeated practice of threat-incongruent trials, and participants are typically not explicitly instructed to avoid threat.

A related ABM method uses the *spatial-cueing task*, which is similar to the visual-probe task except threat and nonthreat cues appear individually on each trial, rather than in pairs (e.g., Bar-Haim, Morag, & Glickman, 2011). Probes appear either in the location just occupied by a cue (valid-cue trial) or in the opposite location (invalid-cue trial). The version of the task used in ABM-threat-avoidance training encourages orienting away from the spatial location of threat cues by more frequent practice of invalid-threat than valid-threat trials; that is, *probes are more likely to appear in the opposite location to threat cues*. Of 32 ABM-threat-avoidance training RCTs considered in this review, 31 employed the visual-probe task and one used the spatial-cueing task for training.

#### Control conditions for ABM-threat-avoidance training

In most studies employing the visual-probe task for ABM training, the control condition typically also uses the visual-probe task with the same threat and nonthreat stimuli, except that probes are equally likely to replace threat and nonthreat cues. This *control-attention-training* (CON-attention-training) condition is designed to control for the effects of multisession practice on the visual-probe task and threat-cue exposure but without modifying AB to threat.

Several ABM-threat-avoidance training studies include additional comparison conditions, such as no-treatment waitlist control (Enock, Hofmann, & McNally, 2014), and attention training using nonthreat cues, such as geometric-attention training, which involves attention training with a contingency between cue and probe locations but without threat-cue exposure (e.g., probes are more likely to replace rectangles than ellipses; Yao, Yu, Qian, & Li, 2015). Another comparison condition is *inverse-ABM* training-toward-threat (Boettcher et al., 2013; Heeren, Reese, McNally, & Philippot, 2012; Heeren, Mogoase, McNally et al., 2015; McNally et al., 2013), which is designed to have the opposite effect of ABM-threat-avoidance training and increase AB toward threat (i.e., using the visual-probe task with probes being more likely to replace threat than nonthreat cues). If AB in orienting to threat plays a causal role in anxiety, inverse-ABM should maintain or enhance anxiety symptoms rather than reduce them; that is, only ABM-threat-avoidance training should reduce both AB to threat and anxiety.

### ABM-positive-search training

ABM-positive-search training uses a visual-search task, which presents an array of pictures on each trial (e.g., 9 pictures arranged in a 3 × 3 array or 16 pictures in a 4 × 4 array). Participants are required to search for a positive/nonthreat target picture embedded among negative/threat distractor pictures (e.g., search for happy face in an angry crowd) (Waters et al., 2013).

An enhanced version of ABM-positive-search training has been developed for anxious children (Waters et al., 2015). It uses multiple positive and calm/nonthreat target-pictures (e.g., children playing, book, armchair) and negative/threat distractor-pictures (e.g., hospital inpatient, aggressive dog, house on fire) to support generalization. It also encourages repeated self-verbalization of attention-search goals to consolidate learning (e.g., “look for good,” “look for calm”) and flexible switching between these goals across different blocks of trials (e.g., look for good in one block, look for calm in another block, and then look for both good and calm cues in a subsequent block of trials). Two ABM-positive-search studies meet the inclusion criteria for this review (Waters et al., 2013; Waters et al., 2015): The former used standard ABM-positive-search procedures, and the latter used the enhanced version.

#### Comparison conditions for ABM-positive-search training

One type of comparison condition involves the same picture-array format as that used in ABM-positive-search training, and participants search for a nonthreat target among nonthreat distractors (e.g., search for a bird among flowers; Waters et al., 2013), which controls for the effect of repeated practice of goal-directed attention-search but not threat-cue exposure. Another comparison condition utilizes a no-intervention waitlist control (Waters et al., 2015).

## Methods Used in AB Assessment

AB change is usually inferred from the difference between pre- and posttraining measures of AB. However, AB measures vary across ABM studies. It is helpful to distinguish between *AB in orienting to threat* (e.g., assessed on visual-probe task) and *AB in threat-distractor interference* (RT-slowing due to task-irrelevant threat; e.g., assessed on modified Stroop task). These AB measures are typically uncorrelated, consistent with different underlying processes (Cisler, Bacon, & Williams, 2009). AB in threat-distractor interference may reflect a combination of automatic interruption of task performance by task-irrelevant threat cues and poor cognitive control of threat-distractor processing (Algom, Chajut, & Lev, 2004; Williams, Mathews, & MacLeod, 1996). Some measures may reflect both types of AB (e.g., nonstandard visual-probe task scores, spatial-cuing task measures; described later), so it may be unclear whether they indicate change in attention responses to threat targeted by ABM-threat-avoidance training (i.e., AB in orienting toward versus away from threat). Thus, it is useful to consider the differing AB measures used in ABM studies before examining the results.

### (i) Visual-probe task

In the version of the task used to assess AB, probes are equally likely to replace threat and nonthreat cues on trials with threat-nonthreat stimulus pairs (i.e., same as CON-attention-training). If an anxious person selectively directs attention to the location of threat cues, they should have faster RTs to probes replacing threat cues (threat-congruent trials) compared with RTs to probes in the opposite location (threat-incongruent trials). The *standard AB score* is the RT-difference between threat-incongruent and threat-congruent trials. Positive values indicate AB in orienting toward threat. Negative values indicate threat avoidance (i.e., faster RTs to probes in the opposite location to threat cues than the same location). AB scores not significantly different from zero indicate no bias. Thus, the *standard AB score reflects the direction of AB in orienting* toward or away from the location of threat, relative to nonthreat cues. The standard AB score is not designed to assess general RT-slowing due to threat-distractor interference, because a threat cue is present on both threat-incongruent and threat-congruent trials.

Some studies also used visual-probe tasks to assess within-session *variation in AB*, reflected by change in AB from beginning to end of each training session (Kuckertz, Amir et al., 2014; Li, Tan, Qian, & Liu, 2008), or within-task fluctuations (Badura-Brack et al., 2015; Clerkin, Magee, Wells, Beard, & Barnett, 2016). It has been suggested that reduction in AB variability (within-task fluctuations) may reflect improvement in attention control as it has been found in anxious individuals during control-attention training, independent of change in standard AB scores (Badura-Brack et al., 2015).

Other ABM studies use *nonstandard visual-probe-task scores*, instead of standard AB scores. Difficulty in orienting away from threat is sometimes inferred from slower RTs to probes replacing neutral cues on threat-neutral trials (threat-incongruent trials) compared with RTs to probes on neutral-neutral cue trials (Carlbring et al., 2012; Kuckertz, Gildebrant et al., 2014; Neubauer et al., 2013). However, this RT-difference compares trials that differ in presence versus absence of threat, so positive values of this score could reflect an interference effect of task-irrelevant threat on RT (threat-related RT-slowing effect, independent of AB in orienting) and/or difficulty orienting away from threat. Thus, it may be unclear from nonstandard AB scores whether they reflect change in AB intended by the ABM training task. A similar issue can apply to interpretation of RTs from the spatial-cuing task (for further discussion of AB measures, see Mogg & Bradley, 2016; Mogg, Holmes, Garner, & Bradley, 2008).

### (ii) Spatial-cuing task

The assessment version of the spatial-cuing task is similar to that used in ABM training, except that the cue-probe contingency is the same for threat and nonthreat cues (i.e., the likelihood of a cue being followed by a probe is the same, irrespective of whether the cue is threat-related or nonthreat). The effect of ABM training on AB can be inferred from the interaction effect on RTs of Training (ABM, CON) × Time (Pre-, Posttraining) × Cue-Valence (Threat, Nonthreat) × Cue-Validity (Valid, Invalid Relationship Between Cue- and Probe-Location). An overall AB index can be calculated (summarizing the interaction effect of Cue-Valence × Cue-Validity on RT), which indicates whether the cue-validity effect of threat cues differs from that of nonthreat cues (Enock et al., 2014; Mogg et al., 2008). It resembles the standard AB index from the visual-probe task but is not often used in ABM studies (Enock et al., 2014; Sigurjónsdóttir, Björnsson, Ludvigsdóttir, & Kristjánsson, 2015).

Analyses of RTs vary across studies, as not all compare invalid-cue and valid-cue trial conditions, and AB is sometimes indexed by the RT difference between invalid-threat versus invalid-nonthreat trials and sometimes by RTs on invalid-threat trials rather than by RT-difference scores (e.g., Amir, Beard, Taylor et al., 2009; Bar-Haim et al., 2011; Liang & Hsu, 2016). Interpretation of RTs can be uncertain; for example, faster RTs on invalid-threat trials at posttraining than pretraining may reflect practice effects on that trial type. Reduction in RT-difference scores (comparing invalid-threat and invalid-nonthreat trials) between pre and posttraining may reflect faster orienting of attention away from threat and/or reduction in the RT slowing effect of task-irrelevant threat cues.

### (iii) Modified Stroop task

In this task, which was used to index AB change by Khanna et al. (2016), AB is inferred from slower color-naming of threat than nonthreat words. This is widely regarded as an AB index of threat-distractor interference. It does not assess AB in spatial orienting toward or away from threat, because the target and distractor information are different attributes of the same stimulus, so the task does not engage spatial orienting.

## Methods Used in Anxiety Assessment

Outcome measures of anxiety vary considerably across studies, which poses a problem for meta-analyses (Cristea et al., 2015), and include self-report questionnaire measures (e.g., generalized anxiety, social anxiety) and standardized clinician-administered diagnostic measures of anxiety disorders and symptom severity. Consistent with previous reviews (e.g., MacLeod & Clarke, 2015), this review focuses on primary outcome measures that were specified a priori or implied in the methods and results (e.g., reduction in self-reported social anxiety symptoms in studies of individuals with high social anxiety; remission of clinician-assessed diagnosis of principal anxiety disorder in individuals with mixed anxiety disorders).

## Findings From ABM Studies Relating to Predictions

### Prediction 1: Effect of ABM on anxiety

#### (i) ABM-threat-avoidance training

*Several studies reported greater anxiety reduction during ABM-threat-avoidance than CON-attention training*. Specifically, eight studies indicated that standard ABM-threat-avoidance training was superior to CON-attention training in reducing anxiety, all of which administered training under experimenter-controlled conditions (i.e., laboratory- or clinic-based settings: Amir, Beard, Burns et al., 2009; Amir, Beard, Taylor et al., 2009; Bar-Haim et al., 2011; Eldar et al., 2012; Hazen, Vasey, & Schmidt, 2009; Kuckertz, Amir et al., 2014; Li et al., 2008; Liang & Hsu, 2016). These studies mostly used the visual-probe task for ABM training (one used the spatial-cuing task; Bar-Haim et al., 2011) with a variety of stimulus types (e.g., pairings of threat-neutral words, disgust-neutral faces, angry-neutral faces, negative-positive faces). One other study by Kuckertz, Gildebrandt et al. (2014), which was home-based, found that anxiety reduction was greater during a combination of ABM-threat-avoidance training and fear-activation (participants were asked to engage in an anxiety-provoking activity before each ABM training session) compared with ABM-threat-avoidance or CON training without fear-activation (data for the latter two conditions were from Carlbring et al. 2012). However, there was no exposure-only condition, so anxiety reduction during the combined condition may possibly be due to fear-activation alone (as repeated exposures to anxiety-provoking activities may reduce anxiety) or its combination with ABM.

However, in most studies, ABM-threat-avoidance training was not more effective than CON-attention training in reducing anxiety between pre- and posttraining. This outcome was found in 23 studies: 15 experimenter-controlled (laboratory-, clinic-, or school-based settings), 7 home-based training, and 1 assessing training in both settings (*experimenter-controlled training*: Badura-Barack et al., 2015, Studies 1 and 2; Britton et al., 2015; Clerkin et al., 2016; Fitzgerald, Rawdon, & Dooley, 2016; Heeren et al., 2012; Heeren, Mogoase, McNally et al., 2015; Khanna et al., 2016; Maoz, Abend, Fox, Pine, & Bar-Haim, 2013; McNally et al., 2013; Pergamin-Hight, Pine, Fox, & Bar-Haim, 2016; Schoorl, Putman, & Van Der Does, 2013; Shechner et al., 2014; Sigurjónsdóttir et al., 2015; Yao et al., 2015; *home-based training*: Boettcher, Berger, & Renneberg, 2012; Boettcher et al., 2013; Boettcher, Hasselrot, Sund, Andersson, & Carlbring, 2014; Carlbring et al., 2012; Enock et al., 2014; Neubauer et al., 2013; Rapee et al., 2013; *laboratory/home comparison*: Carleton et al., 2015).

*Furthermore, in many of these studies, anxiety symptoms reduced irrespective of attention-training condition—that is, during both ABM-threat-avoidance and CON-attention training* (Boettcher et al., 2012, 2013; Britton et al., 2015; Carlbring et al., 2012; Carleton et al., 2015; Clerkin et al., 2016; Enock et al., 2014; Fitzgerald et al., 2016; Heeren et al., 2012; Heeren, Mogoase, McNally et al., 2015; Khanna et al., 2016; McNally et al., 2013; Neubauer et al., 2013; Pergamin-Hight et al., 2016; Schoorl et al., 2013; Shechner et al., 2014). For example, Enock et al. (2014) found greater anxiety reduction in both ABM-threat-avoidance and CON-attention training than a waitlist-control condition. Shechner et al. (2014) found greater anxiety reduction during attention-training (ABM or CON) combined with cognitive behavior therapy (CBT) relative to CBT alone.^3^ Four of these studies also included an inverse-ABM training comparison condition (Boettcher et al., 2013; Heeren et al., 2012; Heeren, Mogoase, McNally et al., 2015; McNally et al., 2013). In Heeren et al. (2012), there was similar reduction in self-reported anxiety between pre- and posttraining during both ABM-threat-avoidance and CON-attention training but not during inverse-ABM training, with further anxiety reduction between posttraining and 2-week follow-up for ABM-threat-avoidance training. In the other three studies, anxiety reduction occurred across all attention-training conditions (ABM, inverse-ABM, CON-attention-training; Boettcher et al., 2013; Heeren, Mogoase, McNally et al., 2015; McNally et al., 2013). One of these studies showed moderate to large improvements in clinical outcome measures across training conditions; moreover, anxiety reduction was unexpectedly *greater* for inverse-ABM training (Boettcher et al., 2013). In addition, two studies unexpectedly found *greater* anxiety reduction during CON-attention-training than during ABM-threat-avoidance training in posttraumatic stress disorder (PTSD) (Badura-Brack et al., 2015).

#### (ii) ABM-positive-search training

In two studies that assessed effects on both anxiety and AB, anxiety reduction was greater for ABM-positive-search than control training or waitlist conditions (Waters et al., 2013, 2015). In these studies, which were both home-based, about a third to a half of children with anxiety disorders were free of their principal anxiety disorder after ABM-positive-search training (50% of those completing ABM versus 8% in control training in Waters et al., 2013, and 42% of those completing ABM versus 8% of waitlist control in Waters et al., 2015).

### Prediction 2: Effect of ABM on AB to threat

To clarify the effect of ABM on AB measures, findings are considered separately for (i) studies of ABM-threat-avoidance training, in which it was more effective in reducing anxiety than comparison conditions; (ii) studies of ABM-threat-avoidance training, in which it was not more effective in reducing anxiety than comparison conditions; and (iii) studies of ABM-positive-search training.

#### (i) Effect of ABM-threat-avoidance training on AB, when there was greater anxiety reduction during ABM than CON-attention training

*These studies also commonly reported changes in performance on AB tasks from pre- to posttraining. However, the nature of these changes varied across studies*. Three studies, which used the visual-probe task to both modify and assess AB, found greater reduction in standard AB scores from pre- to posttraining during ABM than CON training (Amir, Beard, Burns et al., 2009; Eldar et al., 2012; Hazen et al., 2009). In Amir, Beard, Burns et al. (2009), anxious individuals showed no AB before training and showed threat avoidance after ABM training but not after CON-attention training. In Eldar et al. (2012), there was reduction in AB to threat during ABM-threat-avoidance but not CON-attention training in anxious children preselected for exhibiting AB towards threat; AB reduction was found for threat stimuli used during ABM training, which did not generalize to new threat stimuli. Hazen et al. (2009) also found that AB for threat, which was present before training, reduced to no bias during ABM but not CON-attention training but noted that conclusions were limited by small sample sizes at posttraining (*n* = 9 and *n* = 8 in ABM and CON groups, respectively).

Two other studies, which also used the visual-probe task to both assess and modify AB, reported that anxiety reduction during ABM-threat-avoidance training was accompanied by reduction in within-session AB variability (change between beginning and end of each training session) rather than stable change in standard AB scores between pre- and posttraining (Kuckertz, Amir et al., 2014; Li et al., 2008). Neither study showed pretraining AB to threat indexed by standard visual-probe-task scores.

Another study, which also used the visual-probe task to assess and modify AB, compared ABM-threat-avoidance training combined with fear-activation, with ABM-threat-avoidance training without fear-activation and CON-attention-training without fear-activation (Kuckertz, Gildebrant et al., 2014). Nonstandard AB scores on the visual-probe task suggested pretraining AB to threat (slower RTs on threat-present than threat-absent trials), which reduced from pre- to posttraining during ABM with fear activation and CON-only training but not during ABM-only training. Hence, although anxiety reduction was greater in ABM with fear activation than CON-only training, these conditions had a similar effect on AB scores.

Three studies, which found superior anxiolytic effects of ABM-threat-avoidance training (relative to CON-attention training), assessed AB on spatial-cuing tasks (Amir, Beard, Taylor et al., 2009; Bar-Haim et al., 2011; Liang & Hsu, 2016). Although none of these studies showed pretraining AB to threat (i.e., no difference in RTs between invalid-threat and invalid-nonthreat trials), they reported RT changes from pre- to posttraining, which included faster RTs to probes appearing in a different location to threat cues (i.e., faster RTs on invalid-threat trials) following ABM training. Liang and Hsu (2016) found RT changes varied with the exposure duration (100, 500 ms) of stimuli used in training and assessment and suggested that the effect of ABM on RT may reflect changes in both attention control and AB to threat (i.e., results showed a complex five-way interaction that included RT speeding effects on both invalid-threat and invalid-neutral trials that depended on specific stimulus-exposure durations used in AB assessment and ABM training).

#### (ii) Changes in AB to threat, when ABM-threat-avoidance training was not more effective in reducing anxiety than CON-attention training

As noted earlier, many studies found reduction in anxiety symptoms during *both* ABM-threat-avoidance and CON-attention training. Some of these studies also reported reduction in AB measures (which was also independent of type of attention-training method, ABM, CON). Enock et al. (2014) assessed AB change on two tasks (visual-probe and spatial-cuing) and reported that AB reduced during ABM and CON training from no bias to avoidance of disgust faces in socially anxious adults only on the visual-probe assessment task. In Shechner et al. (2014), children with anxiety disorders showed pretraining AB for disgust faces that reduced to no bias during both ABM and CON training. Khanna et al. (2016) used different attentional tasks to assess and modify AB in PTSD (modified Stroop and visual-probe tasks, respectively) and found pretraining AB for combat-related threat words (indexed by threat-distractor-interference effect on RT) reduced during both ABM and CON training, so this AB was no longer evident posttraining. Thus, both anxiety and AB measures reduced during ABM and CON-attention training in these studies (Enock et al., 2014; Khanna et al., 2016; Shechner et al., 2014). By contrast, in Heeren et al. (2012), socially anxious adults showed pretraining AB for angry faces on the visual-probe task, which reduced during ABM-threat-avoidance training (resulting in posttraining AB away from angry relative to happy faces) but did not change during CON or inverse-ABM training, whereas primary social anxiety measures showed a different pattern of results between pre- and posttraining (self-reported anxiety reduced during both ABM-threat-avoidance and CON-attention but not inverse-ABM training).

However, most studies in which anxiety reduced during training, irrespective of condition (ABM-threat-avoidance, CON-attention training, or inverse-ABM), found no change in AB toward threat (e.g., Boettcher et al., 2012, 2013, 2014; Britton et al., 2015; Carlbring et al., 2012; Carleton et al., 2015; Fitzgerald et al., 2016; Heeren, Mogoase, McNally et al., 2015; McNally et al., 2013; Neubauer et al., 2013; Rapee et al. 2013; Schoorl et al. 2013). Most of these studies showed no pretraining AB in anxious individuals, and this was unchanged by training. However, Neubauer et al.’s (2013) results suggested AB *toward* threat at both pre- and posttraining in socially anxious individuals (at each assessment, nonstandard visual-probe-task AB scores reflected slower RTs on threat-incongruent than neutral-neutral trials; standard AB scores were not reported). Conversely, Boettcher et al.’s (2012) results suggested AB *away from threat (avoidance)* at both pre- and posttraining in socially anxious individuals (at each assessment, spatial-cuing-task RTs were faster on invalid-threat than invalid-nonthreat trials).^4^

Two of these studies (i.e., Heeren, Mogoase, McNally et al., 2015; McNally et al., 2013), in which anxiety reduced during training without AB change, also assessed change in attention control during training, using a behavioral measure (Attention Network Task; Fan, McCandliss, Sommer, Raz, & Posner, 2002). In both studies, anxiety reduction was accompanied by improved attention control, irrespective of attention-training method (ABM, CON, or inverse-ABM).

Other studies indicated that anxiety reduction during attention training was accompanied by reduction in AB variability (within-session fluctuation) but not in the standard AB index of orienting to threat on the visual-probe task (Badura-Brack et al., 2015, Studies 1 and 2; Clerkin et al., 2016). In Badura-Brack et al.’s studies, there was greater reduction in PTSD symptoms and AB variability (but not standard AB scores) during CON-attention training compared with ABM-threat-avoidance training, and these changes were interpreted in terms of improvement in attention control. Clerkin et al. (2016) found reductions in AB variability and anxiety symptoms (but not standard AB scores) in socially anxious alcohol-dependent individuals, which occurred during both ABM-threat-avoidance and CON-attention training.

In one study, ABM-threat-avoidance training selectively modified AB to threat but not anxiety (Yao et al., 2015). Yao et al. (2015) compared ABM-threat-avoidance training with two control-training conditions: geometric-attention training (e.g., probes less likely to replace rectangles than ellipses) and control-attention training (no contingency between cue and probe locations). ABM-threat-avoidance training induced an AB away from threat in socially anxious individuals who showed no pretraining AB, and geometric-attention training modified attention to targeted cues. However, the three conditions had no effect on most anxiety measures, although stress-induced speech anxiety reduced from pre- to posttraining irrespective of training condition.

#### (iii) Effect of ABM-positive-search training on AB

Two studies assessed AB change using standard AB scores from the visual-probe task (i.e., different tasks used in ABM training and AB assessment). These showed no significant changes in AB for threat relative to neutral faces during ABM-positive-search training and control conditions (Waters et al., 2013, 2015), with both studies finding no AB for threat either pre- or posttraining. In one study, ABM-positive-search training increased AB for positive relative to neutral faces (Waters et al., 2013); that is, AB for positive faces was found only after ABM-positive-search training and was not evident before training or after control training.

### Prediction 3: Pretraining AB to threat in anxious individuals

*Of studies using standard visual-probe-task scores to assess AB, most did not find AB in orienting toward threat relative to nonthreat stimuli in anxious individuals before training* (e.g., Amir, Beard, Burns et al., 2009; Badura-Brack et al., 2015, Study 2; Boettcher et al., 2013, 2014; Britton et al., 2015; Carleton et al., 2015; Enock et al., 2014; Fitzgerald et al., 2016; Kuckertz, Amir et al., 2014; Li et al., 2008; Maoz et al., 2013; McNally et al., 2013; Pergamin-Hight et al., 2016; Rapee et al., 2013; Schoorl et al., 2013; Waters et al., 2013, 2015; Yao et al., 2015). Eldar et al. (2012) excluded almost half of anxious children originally recruited because they lacked AB for threat, so the sample who underwent attention training all had pretraining AB because of this selection criterion. Only a few studies, which used the standard visual-probe-task index, found the predicted AB to threat in anxious individuals before training: Hazen et al. (2009) found AB for threat relative to neutral words in students with high worry, Heeren et al. (2012) found AB for angry relative to happy faces in socially anxious adults, and Shechner et al. (2014) found AB for disgust relative to neutral faces in children with mixed anxiety disorders.

Studies that assessed pre-training AB from nonstandard visual-probe scores or spatial-cuing tasks showed mixed findings. Comparison of RTs from threat-present (invalid-threat or incongruent-threat trials) and threat-absent (nonthreat) trials included findings of threat-related RT-slowing (Kuckertz, Gildebrant et al., 2014; Neubauer et al., 2013), threat-related RT-speeding (Boettcher et al., 2012), or no RT difference between invalid-threat and invalid-nonthreat trials (Amir, Beard, Taylor et al., 2009; Bar-Haim et al., 2011; Heeren, Mogoase, McNally et al., 2015; Liang & Hsu, 2016) before training. In Sigurjónsdóttir et al. (2015), pretraining AB (assessed on the spatial-cuing task) varied across treatment groups, with some showing AB to threat words and others no bias. One study using the modified Stroop task found a significant interference effect of threat relative to nonthreat words in PTSD sufferers before training (Khanna et al., 2016).

## Discussion: ABM Methods, Outcomes, and Theoretical and Research Implications

As noted in previous reviews (Cristea et al., 2015; MacLeod & Clarke, 2015), ABM has an inconsistent effect on anxiety. It was suggested that failures to find an anxiolytic effect of ABM training can be explained by failures to reduce AB to threat (MacLeod & Clarke, 2015). However, the present review suggests this does not account for mixed findings from studies of ABM-threat-avoidance and ABM-positive-search training, which are considered in turn.

### ABM-threat-avoidance training

The majority of ABM-threat-avoidance training studies did not support the primary prediction; that is, in about two thirds of studies, ABM-threat-avoidance training was not more effective in reducing anxiety than comparison conditions, which in most studies was control-attention training. As noted in previous reviews (Cristea et al., 2015; MacLeod & Clarke, 2015), studies finding a superior anxiolytic effect of ABM-threat-avoidance training were more likely to be laboratory- rather than home-based. The reasons for this are unclear, and suggested explanations are varied. For example, participants have greater contact with experimenters in laboratory/clinic-based than home-based studies and may be more susceptible to experimenter demand effects that may influence self-reported anxiety (Cristea et al., 2015), or participants may be less prone to distraction during laboratory-based training (MacLeod & Clarke, 2015), which may allow them to focus attention more closely on the ABM task (Price et al., 2016). However, effects of experimenter-controlled ABM-threat-avoidance training are not consistent across studies, as the majority of experimenter-controlled studies did not find it to be more effective in reducing anxiety than comparison conditions.

The second main prediction of ABM studies concerns the effect of ABM on AB to threat. Studies that found greater anxiety reduction during ABM-threat-avoidance relative to control training often showed changes in performance on AB assessment tasks. However, interpretation of these results is complicated by methodological issues.

First, their interpretation is made difficult by the use of different AB measures, so it is not always clear that ABM-threat-avoidance training resulted in stable reduction of AB in orienting to threat from pre- to post-ABM training, as intended. Of eight studies that found greater anxiety reduction during standard ABM-threat-avoidance than CON-attention training, three reported correspondingly greater reduction in standard AB scores from the visual-probe task from pre- to posttraining (Amir, Beard, Burns et al., 2009; Eldar et al., 2012; Hazen et al., 2009). However, other studies reported reduction in within-session AB variability (Kuckertz, Amir et al., 2014; Li et al., 2008) or changes in RT indices on spatial-cuing tasks that may reflect reduction in general threat-related RT slowing and/or faster orienting away from threat (Amir, Beard, Taylor et al., 2009; Bar-Haim et al., 2011; Liang & Hsu, 2016). In one study, ABM combined with fear activation was more effective than CON-attention training without fear activation in reducing anxiety (which may have been due to fear activation alone or its combination with ABM) but had a similar effect in reducing nonstandard AB scores (Kuckertz, Gildebrandt et al., 2014).

Second, a widespread methodological problem with most ABM-threat-avoidance training studies is that they assessed AB change using the same or similar probe-based task to that used to modify AB (e.g., as noted by Amir, Beard, Burns et al., 2009; Cristea et al., 2015). Hence, participants may implicitly or explicitly learn a task-specific rule during ABM-threat-avoidance training—that is, *probes are more likely to appear in the opposite location to threat cues*—which would speed RTs on threat-incongruent or invalid-threat trials and result in apparent AB reduction. This task-specific rule applies to both visual-probe and spatial-cuing tasks (i.e., learning this rule would have a similar effect on RTs on threat-incongruent trials on the visual-probe task and invalid-threat trials on the spatial-cuing task), so switching between these tasks for assessment and training would not eliminate this problem. Thus, findings of change in AB measures do not necessarily reflect stable change in AB to threat, beyond the specific demands of the probe-based assessment task.^5^ This methodological issue may also help explain why change in AB to threat is more likely to be found with laboratory-based ABM-threat-avoidance training than home-based training. If laboratory-based training encourages participants to focus attention more closely on the visual-probe training task (Price et al., 2016), this may facilitate learning of the task-specific rule (probes are more likely to occur in a different location to threat cues), resulting in change in performance on a probe-based assessment task that may not necessarily indicate generalized change in AB to threat.

An additional difficulty in interpreting the effects of ABM training on both anxiety and AB measures (given that changes in anxiety and AB measures are assessed at the same time) is in clarifying the mechanisms underlying these changes if they co-occur. That is, anxiety reduction may be a consequence of AB reduction during ABM training. However, ABM training may influence other mechanisms that underlie change in anxiety, such as improvement in attention control (e.g., Chen et al., 2015). If so, AB reduction may be a consequence of improved attention control and/or reduced anxiety (rather than being a cause of anxiety reduction).

This review also indicates that, in many studies, anxiety symptoms reduced to a similar degree during *both* ABM-threat-avoidance and CON-attention training (often without reduction in AB to threat). This observation seems compatible with Price et al.’s (2016) meta-analysis of questionnaire data selected from 11 studies, which indicated that 29% of anxious individuals receiving ABM-threat-avoidance training, and 24% of those receiving CON-attention training, showed improvement in anxiety (indexed by a decrease in social anxiety scores of 30% or more at posttraining relative to pretraining). Repeated findings of anxiety reduction, independent of type of training, suggest a common anxiolytic effect of attention-training. Proposed explanations were tentative as most studies, but not all, lacked non-attention-training comparison conditions. These explanations include anxiolytic effects of positive-outcome expectancies, exposure to threat cues, and increased attention control due to repeated practice of attention-training tasks (Boettcher et al., 2013; Enock et al., 2014; Heeren, Mogoase, McNally et al., 2015; McNally et al., 2013; Price et al., 2016; Shechner et al., 2014).

The role of attention control was suggested by findings that anxiety reduction was accompanied by improved attention control during differing attention-training methods (ABM-threat-avoidance, inverse-ABM, and CON-attention training; Heeren, Mogoase, McNally et al., 2015; McNally et al., 2013). Common features of these training methods include extended practice on attention tasks during exposure to task-irrelevant threat cues, which may promote attention control and ability to ignore threat cues. Thus, the combination of attention training and threat exposure may have an anxiolytic effect (which is common to ABM-threat-avoidance and CON-attention-training), whereas trying to modify the direction of AB in orienting away from threat conveyed no additional anxiolytic benefit in many studies.

As noted earlier, ABM-threat-avoidance training assumes that anxious individuals have a preexisting AB to orient attention toward threat cues relative to nonthreat cues, so it is informative to examine evidence for this prediction. Most ABM studies reviewed here did not find pretraining AB to threat in anxious individuals. Null findings have to be interpreted with caution (e.g., sample sizes tend to be modest; ABs are inferred indirectly from RT data; and RT-based AB measures have low reliability, which poses a challenge for ABM research; MacLeod & Clarke, 2015). However, several researchers remarked on the lack of pretraining AB (e.g., Boettcher et al., 2013; Eldar et al., 2012; Heeren, Mogoase, McNally et al., 2015; Heeren, Mogoase, Philippot et al., 2015; Yao et al., 2015), which is a common finding across ABM studies (see results relating to Prediction 3). Furthermore, other evidence also indicates that anxious individuals do not consistently show AB in orienting toward threat and sometimes show threat-avoidance or no bias (Cisler & Koster, 2010; Dudeney, Sharpe, & Hunt, 2015; Salum et al., 2012; Van Bockstaele et al., 2014; Waters, Bradley, & Mogg, 2014). A meta-analysis published 10 years ago indicated only a small-to-medium effect size for anxiety-related AB to threat on the visual-probe task (*d* = 0.37; Bar-Haim, Lamy, Pergamin, Bakermans-Kranenburg, & van Ijzendoorn, 2007), and more recent reviews conclude that the relationship between anxiety and AB measures is less consistent than widely assumed (Dudeney et al., 2015; Mogg & Bradley, 2016; Van Bockstaele et al., 2014; Waters & Craske, 2016). The appropriateness of ABM-threat-avoidance training has been questioned for anxious individuals who lack AB in orienting toward threat or who may already be threat-avoidant (Eldar et al., 2012; Van Bockstaele et al., 2014).

### ABM-positive-search training

To our knowledge, only two studies have so far assessed effects of multisession ABM-positive-search training on both anxiety and AB in the same sample of anxious individuals, and these showed anxiolytic effects relative to comparison conditions (Waters et al., 2013, 2015). These findings are consistent with other studies of multisession ABM-positive-search training, which were not included in this review because they did not assess effects on both AB and anxiety in the same sample or used unselected participants (Dandeneau et al., 2007; De Voogd et al., 2014; Waters et al., 2016). Together, these findings indicate that this ABM method reduces anxiety and stress symptoms in adults and children in laboratory, school, work, and home settings (Dandeneau et al., 2007; De Voogd et al., 2014; Waters et al., 2013, 2015, 2016). In the latter three studies, ABM-positive-search training was delivered at home to children with anxiety disorders, and about one-third to a half of them were free of their primary anxiety disorder after ABM training. In the latter two studies, therapeutic gains were maintained or improved at 6 months follow-up.

Regarding effects on AB, ABM-positive-search training did not modify AB in orienting to threat in anxious children on the visual-probe task (Waters et al., 2013, 2015). In these studies, different tasks were used to assess and modify AB, and there was also no evidence of AB to threat at pretraining. Thus, this ABM method seems promising in treating anxiety disorders in home settings, and its anxiolytic effect does not seem to depend on reducing AB in orienting to threat.

### Summary of findings

The main findings of this review can be summarized as follows:

- Inclusion of additional ABM studies in this review does not alter conclusions from previous reviews that ABM-threat-avoidance training has an inconsistent anxiolytic effect, relative to control-attention training, and that any additional benefit of this ABM method is largely restricted to experimenter-controlled settings (Cristea et al., 2015; MacLeod & Clarke, 2015). Moreover, the majority of experimenter-controlled studies did not show therapeutic superiority of ABM-threat-avoidance over control training.
- Evidence of AB change during ABM-threat-avoidance training is difficult to evaluate, given methodological issues discussed earlier. Studies use divergent measures (e.g., standard and nonstandard visual-probe task scores, RTs on spatial-cuing tasks, modified Stroop scores, AB variability scores), which reflect differing manifestations of AB (e.g., AB in orienting toward threat, AB in threat-distractor interference, unstable AB fluctuating between threat vigilance and avoidance). Moreover, it is uncertain whether changes in AB measures from pre- to post-ABM-threat-avoidance training reflect specific experimental task demands and/or more general change in AB to threat. This prevents clear conclusions regarding whether the anxiolytic effect of ABM-threat-avoidance training depends on stable reduction of AB in orienting toward threat, as targeted by this ABM method.
- In most ABM studies, anxious individuals did not show preexisting AB in orienting toward threat (a fundamental assumption of ABM-threat-avoidance training).
- Anxiety symptoms often reduced during both ABM-threat-avoidance and control-attention training (sometimes with moderate-to-large effect sizes, and often without change in AB to threat).
- Some studies indicate that anxiety reduction during attention training is accompanied by improvement in attention control.
- Multisession ABM-positive-search training seems promising in reducing anxiety, including when used in home settings, and its anxiolytic effect does not depend on reducing AB in orienting to threat.

Together, these findings indicate that anxiolytic effects of ABM and attention training do not depend on reduction of preexisting AB in orienting toward threat in anxious individuals. Hence, their anxiolytic effects are likely to involve other mechanisms.

### Theoretical considerations

To interpret these findings, it is helpful to consider differing cognitive models of anxiety and their implications for ABM training. As noted earlier, ABM-threat-avoidance training is supported by a cognitive view of anxiety, which proposes that anxious individuals are characterized by an enhanced automatic AB toward threat, which plays a causal role in anxiety (e.g., Williams, Watts, MacLeod, & Mathews, 1988). This view further assumes that low-anxious individuals are characterized by attentional avoidance of threat. Hence, it predicts that an intervention that reduces preexisting AB toward threat in anxious individuals and instead makes them threat avoidant should reduce anxiety. This view supports the use of implicit training procedures, because the anxiety-related AB toward threat is assumed to operate automatically and outside awareness, so it may be modified primarily by habit change via repeated practice, rather than by involving explicit controlled processes (Bar-Haim, 2010).

**Fig. 1.**
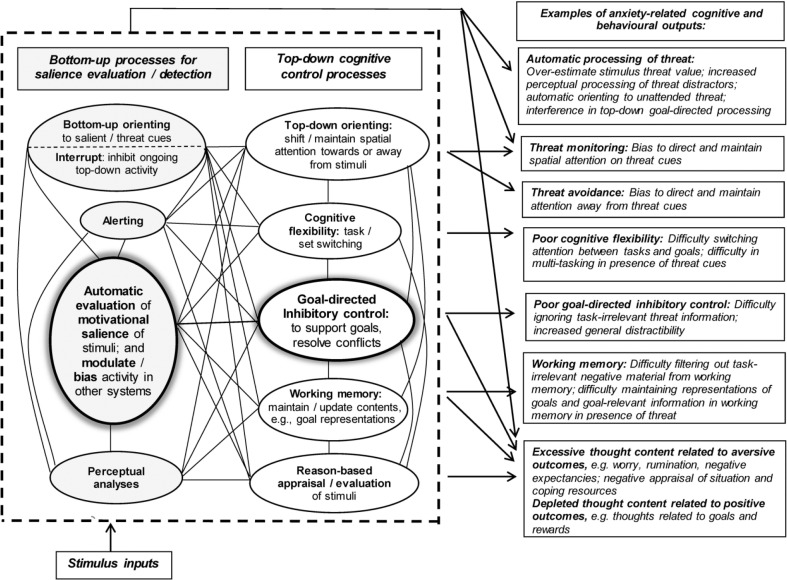
Bottom-up and top-down cognitive systems, and their component functions, which may contribute to anxiety and ABs to threat (adapted from Mogg & Bradley, 2016).

However, most cognitive models of anxiety propose that both bottom-up (automatic) and top-down (controlled) processes play an important role in anxiety and attention responses to threat (e.g., Bar-Haim et al., 2007; Beck & Clark, 1997; Cisler & Koster, 2010; Eysenck, Derakshan, Santos, & Calvo, 2007; Lonigan, Vasey, Phillips, & Hazen, 2004; Mogg & Bradley, 1998, 2016; Waters & Craske, 2016; Williams, Watts, MacLeod, & Mathews, 1997). Models of anxiety, attention, and cognitive control together point to a multicomponent framework of bottom-up processes, which support rapid evaluation and detection of threat, and top-down control processes, which support goal-directed activity and emotion regulation (see Fig. 1; for a review, see Mogg & Bradley, 2016).

According to recent evidence-based models of attention and cognitive control, top-down functions include *goal-directed inhibitory-control* (which is a core component underlying a wide range of top-down control functions), cognitive flexibility, maintaining and updating goals in working memory, reason-based appraisal, and controlled attention orienting (Diamond, 2013; Miyake & Friedman, 2012; Petersen & Posner, 2012). These top-down functions typically operate in a unified manner to support goal-directed activity (Duncan, 2010).

Bottom-up functions include *automatic stimulus-salience evaluation*, alerting, automatic interrupt/inhibition of ongoing goal-directed activity, and automatic attention orienting to threat (Corbetta & Shulman, 2002; Pessoa & Adolphs, 2010; Petersen & Posner, 2012). The stimulus-salience-evaluation mechanism plays a key role in automatically evaluating the motivational salience of stimuli and modulating other processes to prioritize salient stimuli (Pessoa & Adolphs, 2010); for example, it triggers automatic interruption of goal-directed activity and biases attention orienting toward potential threat cues. Reactivity of this salience-evaluation system to threat cues and its influence on other processes (e.g., on attention orienting) can be opposed by top-down cognitive control. The latter supports task-related and emotion-regulation goals and may suppress AB to threat or trigger threat avoidance (Cisler & Koster, 2010; Mogg & Bradley, 1998; Williams et al., 1997).

According to this framework, both excessive anxiety and ABs to threat reflect imbalance between these bottom-up and top-down systems. This imbalance gives rise to differing manifestations of anxiety-related ABs in attention orienting, such as initial orienting to threat, maintained attention on threat, and threat avoidance, and AB in threat-distractor interference. Thus, AB to threat is not a unitary construct, and its manifestations reflect differing combinations of processes (Fig. 1). For example, AB in threat-distractor interference can be observed on tasks that do not involve attention orienting (e.g., modified Stroop task), and this AB index is typically uncorrelated with measures of AB in orienting to threat (Cisler et al., 2009). ABs to threat are influenced by multiple variables (e.g., stimulus threat value, situational stress, comorbidity between anxiety, fear and trauma symptoms; see Mogg & Bradley, 2016, for a review). Variation in ABs may be partly explained by anxious individuals recruiting top-down cognitive control processes that oppose threat processing to support task-focused goals (e.g., suppression of AB in high-anxious individuals under stress; Amir et al., 1996; Williams et al., 1997) or emotion-regulatory goals (e.g., threat avoidance to reduce subjective discomfort elicited by threat cues; Cisler & Koster, 2010; Mogg & Bradley, 1998). ABs in orienting to threat may also rapidly fluctuate between threat vigilance and avoidance (Badura-Brack et al., 2015; Mogg & Bradley, 1998, 2016; Zvielli, Bernstein, & Koster, 2014). Hence, anxiety-related ABs in orienting to threat may be unstable, which may make them difficult to detect on the visual-probe task and modify in ABM studies.

Excessive anxiety may arise from a combination of inadequate top-down goal-directed inhibitory control and hyperreactivity to threat cues of the bottom-up threat-salience-evaluation mechanism, resulting in its overdominant bias on other processes. This view not only integrates proposals from prior models of anxiety but also has implications for ABM research. For example, methods that combine extensive practice on attention tasks with threat-cue exposure may have anxiolytic effects by strengthening top-down cognitive control, in particular goal-directed inhibitory control over processing task-irrelevant threat, which opposes activity of the bottom-up threat-salience-evaluation mechanism and its influence on other cognitive processes. As top-down cognitive-control functions operate in a unified manner to support goals (Duncan, 2010), interventions that promote coordinated functioning of multiple top-down functions during exposure to threat cues may be more effective in correcting imbalance between top-down and bottom-up systems than interventions that target isolated functions (e.g., automatic orienting toward threat).

This theoretical view may help explain why anxiety reduction often occurs during both ABM-threat-avoidance and control-attention training, as both methods combine extended practice on attention tasks and threat-cue exposure, which together may enhance top-down cognitive control and effortful threat-distractor inhibition. Encouraging anxious individuals to orient attention away from the location of threat cues may convey little additional benefit, if modifying attention control (including threat-distractor inhibition) plays a more important role in mediating the anxiolytic effect of ABM, rather than modifying direction of orienting toward threat (Heeren, Mogoase, McNally et al., 2015).

Also, in relation to the framework outlined in Figure 1, the enhanced version of ABM-positive-search training (Waters et al., 2015, 2016) may recruit multiple top-down cognitive-control functions during exposure to a diverse range of threat cues, including effortful *goal-directed inhibitory control* of threat-distractor processing; *controlled orienting* toward positive and nonthreat cues and away from negative and threat cues; *cognitive flexibility* in switching between adaptive goals: “look for good,” “look for calm”; *maintaining adaptive attention-search goals in working memory* by verbal rehearsal of them; and *task-related appraisal* of diverse threat cues as goal-irrelevant. Thus, although this method is a variant of ABM training, because it trains attention search for positive/nonthreat versus negative/threat stimuli, its anxiolytic effect may depend on influencing multiple processes. Also, it is potentially suitable for all anxious individuals, irrespective of whether or not they show preexisting AB in orienting toward or away from threat, or unstable AB (for further discussion of ABM-positive-search and ABM-threat-avoidance training methods, see Mogg & Bradley, 2016).

### Implications for ABM research

To avoid some of the challenges and pitfalls faced by research into ABM-threat-avoidance training due to rapid publication of mostly small-scale studies with varying methodology across studies (see Cristea et al., 2015), ABM-positive-search training (and any newly emerging ABM approaches; see review by Mogg & Bradley, 2016) requires carefully controlled RCTs against key comparison conditions. This entails adequately powered RCTs using large samples of individuals with anxiety disorders; a prespecified, reproducible protocol for the intervention; and intention-to-treat analyses to facilitate cost-benefit evaluation (Andrews, Cuijpers, Craske, McEvoy, & Titov, 2010; Cristea et al., 2015; Washington State Institute for Public Policy [WSIPP], 2016a). To evaluate the effects of ABM, such RCTs require thorough assessments of anxiety (and related symptom and process measures; e.g., depression, treatment satisfaction), ABs, and other attention variables such as attention control. This review highlights the importance of the following key issues for ABM research.

#### Choice of ABM methods

One important goal of ABM research is to develop an effective intervention that can be delivered easily and inexpensively to clinically anxious individuals in their homes and other settings such as primary care. Given the disappointing findings for ABM-threat-avoidance training, several other novel methods have been proposed, in addition to ABM-positive-search training (e.g., Dennis & O’Toole, 2014; Notebaert, Clarke, Grafton, & MacLeod, 2015; Zvielli, Amir, Goldstein, & Bernstein, 2015; see review by Mogg & Bradley, 2016).^6^ However, none of these novel methods has yet been used with anxiety disorders.

Decisions regarding which ABM methods are likely to be clinically effective in reducing anxiety, and consequently require further evaluation, need to be based not only on empirical evidence (e.g., which interventions show promise in reducing clinical and nonclinical anxiety in adults and children in both experimenter-controlled and home settings) but also guided by evidence-based cognitive models of anxiety (e.g., which processes contribute to anxiety and how they might be modified). For example, recent models of anxiety predict that ABM methods that recruit multiple top-down goal-directed cognitive-control processes to oppose threat processing will be more effective in reducing anxiety than methods targeting one specific bias in direction of orienting toward threat (e.g., Mogg & Bradley, 2016; Waters & Craske, 2016). Hence, these models predict that enhanced ABM-positive-search training will be more effective in reducing anxiety than conventional ABM-threat-avoidance training.

#### Choice of comparison conditions

Decisions about control conditions employed in ABM research should be guided by cognitive models of anxiety, empirical evidence, methodological considerations, and specific research goals. For example, the use of control-attention training (combining attention-task practice and threat-cue exposure) as the main comparison condition for ABM-threat-avoidance training was justified by the theoretical view that an AB in attention orienting toward threat plays a key causal role in anxiety. Control-attention training was not predicted to reduce anxiety, because it was not designed to modify this specific AB in direction of orienting to threat cues. However, contrary to this view, many studies found that anxiety reduces during attention-control training (as well as during other comparison conditions, such as inverse-ABM). As noted earlier, such findings may be explained by a differing theoretical view, which proposes that dysfunction in multiple processes (including top-down attention control and threat-distractor inhibition) contributes to anxiety and differing manifestations of ABs and may be modified by both active ABM and control-attention training.

Thus, the choice of comparison condition depends on the evidence-based theoretical rationale for the ABM method, as well as the specific study aims, as differing comparison conditions are useful for different research purposes. If the study aims to evaluate the therapeutic efficacy of ABM for anxiety disorders, it is helpful to include a comparison condition that is relevant to its potential real-life clinical application (e.g., in primary care), such as minimal-intervention psychoeducation or no-intervention waitlist conditions (e.g., Enock et al., 2014; Waters et al., 2015; see also Khanna et al., 2016, who discuss advantages of a no-intervention condition). Such conditions control for effects of repeated assessment and provide clinically useful information that facilitates cost-benefit analyses for health care providers (Andrews et al., 2010; National Institute for Health and Care Excellence [NICE], 2011; WSIPP, 2016a, 2016b). Comparisons with waitlist and minimal-intervention conditions inform whether ABM training may be a useful treatment at an early stage of a stepped-care approach for anxiety disorders, prior to later stages involving more intensive therapist-delivered interventions, which may not subsequently be required if ABM training proves effective.

However, such comparisons do not control for expectancy effects or provide information about specific features of ABM training that may convey therapeutic benefit, which need to be addressed by including additional comparison conditions (e.g., controlling for effects of repeated practice on attention tasks, independent of threat-cue exposure). Establishing whether an intervention is effective in treating anxiety requires a systematic approach comparing it with a series of different conditions to examine specific issues (Lohr, Lilienfeld, & Rosen, 2012). For ABM methods, this approach includes (a) comparison with waitlist or minimal-intervention conditions, as discussed earlier; (b) comparison with control training conditions to identify key ingredients of ABM training that may contribute to its efficacy, as predicted by cognitive models of anxiety (e.g., extended attention training, threat-cue exposure), and also to control for nonspecific-treatment effects; (c) comparing laboratory- and home-delivered ABM, to evaluate whether it has widespread applicability; (d) comparison of ABM training with other potential first-line interventions, such as computer-delivered CBT, which may also be used in primary care within a stepped-care approach (Craske et al., 2009; NICE, 2011; Richards, Richardson, Timulak, & McElvaney, 2015; WSIPP, 2016b); and (e) comparing conventional treatments (e.g., face-to-face CBT, or medication) with and without concurrent ABM, to evaluate whether ABM augments their anxiolytic effects. Studies may also benefit from using more than one comparison condition to assist interpretation of results, as discussed earlier (e.g., to assess whether ABM and the key comparison condition, such as control-attention training, have anxiolytic effects relative to no- or minimal- intervention).

Recent reviews of evidence from such comparisons have led to the conclusion that conventional ABM-threat-avoidance training is not clinically useful (e.g., Cristea et al., 2015; MacLeod & Clarke, 2015; present review). However, the clinical effectiveness of other ABM methods, such as multisession ABM-positive-search training, remains to be established.

#### Choice of attention measures

To clarify the cognitive mechanisms underlying the anxiolytic effects of those ABM training methods that are effective, it is important to assess their effects on ABs and other attention variables. Anxiolytic effects of ABM may relate to improvements in attention control and threat-distractor inhibition, which are distinct from modifying the direction of AB in orienting away from threat. However, few multisession-ABM studies have included measures of attention control (as in Heeren, Mogoase, McNally et al., 2015; McNally et al., 2013) or AB in threat-distractor interference (as in Khanna et al., 2016). Also, as noted earlier, interpretation of findings of AB change in previous studies of ABM-threat-avoidance training is complicated by methodological problems, such as diverse AB measures (some of which are difficult to interpret, as discussed earlier), and uncertainty regarding whether change in AB to threat (if found) generalized beyond probe-based tasks to other tasks.

Thus, several issues influence the choice of attention measures in ABM studies:

- ABM studies should assess changes in attention variables that are predicted by cognitive models of anxiety to underlie the therapeutic effect of ABM. Thus, it would be helpful to include measures of AB in attention orienting to threat, AB in threat-distractor inference, AB variability, and attention control.
- The choice of AB measures should allow evaluation of whether change in AB generalizes beyond the training paradigm, by including a task that not only uses different stimuli but also does not share the same task demands and rules as the ABM training task (e.g., assess AB on modified Stroop or flanker tasks before and after probe-based ABM; assess AB on the visual-probe task before and after visual-search-based ABM).
- Comparison of findings across ABM studies would be facilitated by more consistent use of established assessment methods (e.g., standard index of AB in orienting to threat from the visual-probe task with angry-neutral face pairs).
- Measures of AB variability (e.g., on the visual-probe task; Badura-Brack et al., 2015; Zvielli et al., 2014) should be accompanied by measures of RT variability, which is also linked with psychopathology and poor attention control (e.g., Bastiaansen, van Roon, Buitelaar, & Oldehinkel, 2015; Weissman, Roberts, Visscher, & Woldorff, 2006) and may contribute to RT-based measures of AB variability (Kruijt, Field, & Fox, 2016).
- When assessing attention control, it is helpful to use both objective and subjective measures (e.g., Attention Network Task, Fan et al., 2002; and Attention Control Scale, Derryberry & Reed, 2002; Melendez, Bechor, Rey, Pettit, & Silverman, 2016; see Heeren, Mogoase, McNally et al., 2015; McNally et al., 2013).
- Eye-tracking and neural measures of AB and attention control can be included to complement RT-based measures and may provide a clearer delineation of component processes (Britton et al., 2013; Chen et al., 2015; Holmes, Mogg, de Fockert, Nielsen, & Bradley, 2014; Mogg & Bradley, 2016; Waters & Craske, 2016; White et al., 2016).

To take account of these issues, each ABM study should ideally use multiple attention measures. Also, when assessing ABs, it is useful to consider the extent to which differing measures may possess trait- and state-like characteristics, which would influence their psychometric properties (Zvielli et al., 2014). For example, poor reliability of measures of AB in orienting to threat may not only reflect measurement error but also indicate that this index does not have strong stable trait-like characteristics. ABs may have both state- and trait-like characteristics and reflect an individual’s current motivational/goal priorities that are dominant in a particular situation (e.g., to automatically detect unattended threat; to maintain attention on threat to allow more detailed evaluation; to avoid attending to threat cues to minimize subjective discomfort; or to suppress threat-related AB to support task-focused processing), and these priorities may in turn depend on multiple task-related and individual-difference variables (for further discussion of variables influencing ABs, see Mogg & Bradley, 2016).

## Concluding Comments

Key features of this review are that (a) it considers empirical findings from 34 RCTs examining effects of multisession ABM on both AB and anxiety in high-anxious individuals; (b) it distinguishes between different ABM training methods (ABM-threat-avoidance, ABM-positive search) and manifestations of AB (e.g., AB in orienting toward versus away from threat, AB in threat-distractor interference, AB variability; see Mogg & Bradley, 2016, for further discussion of differing ABs); (c) it challenges the core theoretical assumption underlying ABM-threat-avoidance training that anxious individuals are characterized by enhanced AB in orienting to threat (as assessed by visual-probe and spatial-cueing tasks) and that this AB plays a primary causal role in anxiety; (d) it concludes that the anxiolytic effects of ABM and attention training do not depend on reducing preexisting AB in orienting attention toward threat in anxious individuals (as anxious individuals often do not show this AB, and anxiety reduction is not consistently accompanied by reduction of this AB); (e) it highlights the role of multiple top-down and bottom-up mechanisms, which are proposed by cognitive models to underlie anxiety and which may contribute to anxiolytic effects of ABM, such as goal-directed attention control and threat-distractor inhibition (Mogg & Bradley, 2016); and (f) it considers the implications of theoretical and empirical developments in ABM research—for example, for choice of ABM training methods, comparison conditions, and attention measures—and importantly, it provides specific recommendations for advancing AB and ABM research.

Disappointing conclusions regarding the poor clinical utility of ABM-threat-avoidance training may not apply to other ABM methods, such as ABM-positive-search training, which may prove useful in offering home-based computer-delivered treatment for anxiety disorders. A limitation is the small number of studies of ABM-positive-search training that have so far assessed its effects on both AB and anxiety symptoms in anxious individuals. However, preliminary studies indicate that this method is effective in reducing nonclinical and clinical anxiety in both experimenter-controlled and home-based settings and thus merits further evaluation (Dandeneau et al., 2007; De Voogd et al., 2014; Waters et al., 2013, 2015, 2016).

This review also identifies methodological issues (e.g., ABM methods, comparison conditions, AB assessment) that influence the interpretation of findings from ABM studies. For example, it is important to clarify whether any AB change is specific to the experimental context or reflects more meaningful (ecologically valid) change in attention responses to threat that generalize to other tasks or situations (e.g., using dissimilar AB tasks at pre- and posttraining). Also, it is helpful to examine whether generalization of ABM effects is enhanced by specific attention-training variables, such as using large stimulus arrays of diverse pictorial cues, in which goal-relevant nonthreat targets are simultaneously presented with multiple threat distractors, and encouraging explicit rehearsal of adaptive attention-search goals, such as self-instruction to look for good and calm stimuli (e.g., Waters et al., 2015, 2016).

Another issue that complicates evaluation of effects of ABM on AB and anxiety is dissemination bias (for discussion, see Cristea et al., 2015; Song, Hooper, & Loke, 2013). Potential sources of bias include publication bias (“file-drawer” problem) and outcome-reporting bias (“cherry-picking”), which may influence the ratio of significant to null findings in published reports. For example, publication bias, which favors reports of novel significant findings, leads to underrepresentation in journal articles of studies finding null differences between ABM and control conditions. Outcome-reporting bias may occur in a study where multiple outcome measures are available (e.g., multiple anxiety measures, or differing RT indices of AB) and favors reporting of those that show significant effects. Thus, it is helpful to consider such potential sources of bias when interpreting results from ABM studies (Cristea et al., 2015). Preregistration of RCTs with details of primary anxiety and AB outcome measures is also helpful (Emmelkamp, 2012), together with more consistent use of AB measures in ABM studies.

Furthermore, the review highlights the importance of considering divergent cognitive views of anxiety, which may explain why differing ABM methods vary in efficacy. According to recent cognitive models of anxiety, multiple top-down and bottom-up processes underpin anxiety, emotion regulation, and attention responses to threat. Thus, the therapeutic efficacy of ABM methods may depend on modifying multiple processes within these cognitive systems (e.g., goal-directed inhibitory control, effortful threat-distractor inhibition, automatic and controlled attention-orienting, attention flexibility, evaluation of motivational salience and goal relevance of threat cues, maintaining adaptive attention-search goals in working memory), rather than solely targeting one manifestation of AB—that is, attention orienting toward threat, which is not consistently found in anxious individuals. Thus, further systematic research is required to determine which ABM methods are effective in reducing anxiety, while taking account of existing empirical findings and methodological and theoretical issues that influence their interpretation.

## Notes

1. Eighteen of 22 multisession studies in MacLeod and Clarke’s (2015) Table 1 used an RCT design, high-anxious individuals, and ABM training with threat and nonthreat stimuli. The other four studies differed in several respects: One was designed to increase AB for positive stimuli and did not use threat cues in training (Britton et al., 2013); another used a case-series rather than RCT design (Schoorl et al., 2014); and two studies used unselected rather than high-anxious participants (ABM-threat-avoidance training, See, MacLeod, & Bridle, 2009; ABM-positive-search training, De Voogd et al., 2014).

2. The selection criteria used in recent systematic and quantitative reviews vary according to specific review aims and methods (e.g., type of anxiety under consideration; inclusion of single- and multisession ABM; need for effect size data in meta-analyses). These included a systematic review of 10 ABM studies in child and adolescent anxiety that included nonrandomized controlled studies (Lowther & Newman, 2014); meta-analysis of 22 comparisons from single- and multisession ABM RCTs in subclinical and clinical anxiety as part of a larger review (Mogoase et al., 2014); systematic review and meta-analysis of 15 single- and multisession ABM RCTs in subclinical and clinical social anxiety (Heeren, Mogoase, Philippot et al., 2015); meta-analysis of single- and multisession RCTs of cognitive bias modification in anxiety and depression including 16 comparisons for ABM in subclinical and clinical anxiety (Cristea et al., 2015); meta-analysis of 11 multisession probe-based ABM RCTs in clinical anxiety (Linetzky, Pergamin-Hight, Pine, & Bar-Haim, 2015); and meta-analysis of pooled patient-level data from multisession ABM RCTs of subclinical and clinical anxiety, using self-report anxiety data from 11 studies and AB data from 10 studies (Price et al., 2016).

3. Three other studies compared ABM- and CON-attention training, each combined with CBT, and found similar anxiety reduction in both training conditions (Boettcher et al., 2014; Rapee et al., 2013; Sigurjónsdóttir et al., 2015); these studies did not include a CBT-only comparison condition, so anxiety reduction may be explained by CBT effects.

4. Of studies that found anxiety reduction during both ABM and CON training, not all reported summary statistics—that is, means and *SD*s—for AB scores separately at *both* pre- and posttraining (e.g., Carlbring et al., 2012; Clerkin et al., 2016; Pergamin-Hight et al., 2016), and AB changes were difficult to interpret when treatment conditions had small sample sizes and varied in pretraining AB (Sigurjónsdóttir et al., 2015).

5. One ABM-threat-avoidance training study did not have this methodological problem as it used the visual-probe task with angry faces for training (ABM-threat-avoidance and CON-attention training) and the modified Stroop task with threat words for AB assessment (Khanna et al., 2016). Anxiety and AB reduced from pre- to posttraining in both training conditions; that is, AB reduction was not attributable to ABM. Other ABM-threat-avoidance training studies, which examined generalization of AB change to non-probe-based assessment tasks, such as modified flanker or visual-search tasks, failed to find evidence of generalization (e.g., studies of single-session ABM training in unselected participants: Grafton, Mackintosh, Vujic, & MacLeod, 2013; Van Bockstaele, Salemink, Bögels, & Wiers, 2015).

6. These methods vary considerably in rationale and methodology. Dennis and O’Toole (2014) developed a computer-game format of ABM-threat-avoidance training, including colorful displays, animated characters (angry and mildly positive cartoon faces), sound effects, and trial-by-trial feedback to improve participant engagement and learning of threat avoidance. Notebaert et al.’s (2015) “attend-happy” version of ABM also has a gamified format (resembling a card game); each trial presents two happy and two angry faces, and participants make a person-identity-matching judgement for the two happy faces (i.e., are the happy faces the same person, or two different people?). It differs from standard ABM-threat-avoidance training by including explicit instructions to attend to the emotional content of the faces and to respond to positive target-cues and requiring participants to effortfully ignore threat distractors that are presented simultaneously with the positive targets. Zvielli et al.’s (2015) method is based on the premise that ABs in orienting to threat are unstable in anxiety (i.e., fluctuate between threat vigilance and avoidance). It provides trial-level feedback during a visual-probe task to encourage stable responding across conditions (threat-congruent, threat-incongruent, and neutral-neutral trials) and improve cognitive control over unstable ABs. The evaluation of these novel methods is so far limited to exploratory studies of effects of single-session training (rather than RCTs of multisession ABM training with high-anxious individuals), and consequently these studies did not meet selection criteria for inclusion in this review.
